# Efficient Regulation of the Cross-Linking Structure in Polyurethane: Achieving Outstanding Processing and Mechanical Properties for a Wind Turbine Blade

**DOI:** 10.3390/polym16020235

**Published:** 2024-01-15

**Authors:** Zijin Jiang, Lingtong Li, Luoping Fu, Gaohu Xiong, Hong Wu, Shaoyun Guo

**Affiliations:** 1 The State Key Laboratory of Polymer Materials Engineering, Polymer Research Institute of Sichuan University, Chengdu 610065, China; jiangzijin@dongfang.com (Z.J.); lingtongli1994@163.com (L.L.); nic7702@scu.edu.cn (S.G.); 2 Sichuan Dongshu New Materials Co., Ltd., Deyang 618000, China; fuluoping@outlook.com (L.F.); xionggaohu@dongfang.com (G.X.)

**Keywords:** polyurethane, cross-linking, processing, mechanical property, wind turbine blade

## Abstract

Although epoxy resin has been extensively used in the field of wind turbine blades, polyurethane has attracted much attention in recent years, due to its potential value of better fatigue resistance, lower processing viscosity and higher strength than epoxy resin blades. Herein, we construct a dense cross-linking structure in polyurethane (PU) based on different amounts of hydroxypropyl methacrylate (HPMA) with low processing viscosity and excellent mechanical properties. By increasing the content of HPMA, the thermal stability of PU is enhanced, but the micro-morphology does not change significantly. When the content of HPMA is 50 g (in 200 g copolymer), the PU sample PH-50 exhibits a viscosity of 70 MPa·s and a gelation time of 120 min at 25 °C, which is sufficient to complete processes like pouring and filling. By post-curing the PH-50 at 80 °C for 2 h, the heat distortion temperature can reach 72 °C, indicating the increase of temperature resistance. The PU copolymers also have excellent mechanical and dynamic thermo-mechanical properties due to the cross-linking structure between PU chains and poly-HPMA chains. Additionally, the PU copolymer has excellent compatibility with various glass fiber fabrics (GFF), showing a good match in the vacuum infusion experiment and great properties in the mechanical test. By compounding PH-50 with GFF, the composite with high strength is easily prepared for a wind turbine blade in various positions. The tensile strengths of the composites are all over 1000 MPa in the 0° direction. Such composites are promising for the future development of wind turbine blades that meet the stringent requirements for outstanding processing and mechanical properties.

## 1. Introduction

Fossil energy has caused increasingly serious energy consumption and environmental pollution in recent years [1,2]. Wind energy, as an environmentally friendly energy source, has received widespread attention and undergone vigorous development [3]. Currently, wind power has become the main force of clean energy generation [4]. Although the installed capacity of wind power is on a growth trend, the manufacturing of wind turbine blades is still difficult, complex and expensive [5,6]. Wind turbine blades vary in size and do not use exactly the same materials [7,8]. Manufacturing such a large installation requires a lot of reinforcing fibers, core materials, matrix resins, bonding adhesives and other materials [9]. Among these materials, the performance of the matrix resins greatly determines the processing and mechanical properties of the wind turbine blade [10].

Thermoset polymers have been widely used in aviation, aerospace, automotive and other structural materials because of their excellent physicochemical properties [11,12]. Recently, epoxy resin has become one of the most important materials in the field of wind turbine blades due to its structural stability, light weight, high strength, high modulus, low shrinkage and good dimensional stability [13,14,15]. In contrast, PU has the potential benefit of better fatigue resistance, lower viscosity, higher strength, higher modulus and lower shrinkage than epoxy resin, and these benefits have attracted attention in the wind turbine blades field [16,17,18]. Despite the potentials of PU, its poor processing characteristics have limited its development in recent years. Specifically, the isocyanates that make up PU are highly reactive with polyols [19,20]. The short gelation time has a significant influence on the structure in the infusion and curing process, which ultimately leads to PU having different mechanical properties [21]. Therefore, it is still necessary to find an efficient method to prolong the gelation time of PU, meeting the processing needs and obtaining dense cross-linking structure to expand the practical application.

There are many ways to extend the gelation time of PU to obtain the dense cross-linking structure, such as adjusting the ambient temperature, increasing the molecular weight of the soft segment and adding some materials to slow down the reaction [22,23]. Among them, reacting PU prepolymers with some special monomers should be an effective option [24]. The monomer used for the reaction should have polymeric groups and the ability to react with isocyanate. The viscosity of PU prepolymer can be significantly reduced due to the low viscosity monomers at room temperature. With the reaction proceeding and the temperature rising, the dense cross-linking structure of PU chains forms an enhanced network structure. Up to now, there are few reports on extending the gelation time of PU to obtain a dense cross-linking structure in PU by incorporating radical polymerization.

In this work, we construct a dense cross-linking structure in polyurethane (PU) based on different amounts of hydroxypropyl methacrylate (HPMA) to achieve low processing viscosity and excellent mechanical properties. The viscosity, gelation time and cross-linking structure of the prepolymer can be regulated by adjusting the amount of HPMA monomer. We found that the prepolymer PH-50 has a low viscosity and a long gelation time at 25 °C, which is sufficient for processes such as pouring or filling. After the pouring and curing processes, the PU copolymers shows excellent mechanical and dynamic thermo-mechanical properties due to the cross-linking structure between the polyurethane and poly-HPMA chains. In addition, the PU samples show good compatibility with various glass fiber fabrics (GFF) in vacuum infusion experiments. The PU/GFF composites have high strength and modulus, which makes them suitable for wind turbine blades under various operating conditions. This PU/GFF composite would have great potential for developing wind turbine blades that meet the stringent requirements for outstanding processing and mechanical properties in the future.

## 2. Materials and Methods

### 2.1. Materials

Polymerized isocyanate MDI (PM200) was purchased from Wanhua Chemical Polyurethane Co., Ltd. (Yantai, China). Polyether polyols DL400 and MN500 were purchased from Bluestar Dongda Co., Ltd. (Zibo, China) and Zibo Dexin Federal Chemical Co., Ltd. (Zibo, China), respectively. Hydroxypropyl methacrylate (HPMA) was purchased from Shanghai Dongtu Chemical Import & Export Co., Ltd. (Shanghai, China). Defoamer BYK088 was purchased from Shanghai HaiyiTrading Company (Shanghai, China)). The accelerator cobalt naphthenate (CN), initiator methyl ethyl ketone peroxide (MEKP) and chelator citrate were purchased from Aladdin Reagent Co., Ltd. (Shanghai, China). Glass fiber fabrics (E6-UD1200, E7-UD1250, E8-UD1250 and E7-UD650) for vacuum infusion were purchased from Zhejiang Hengshi Fibre Base Co., Ltd. (Jiaxing, China). 

### 2.2. Preparation of PU Copolymers

**Figure 1:**
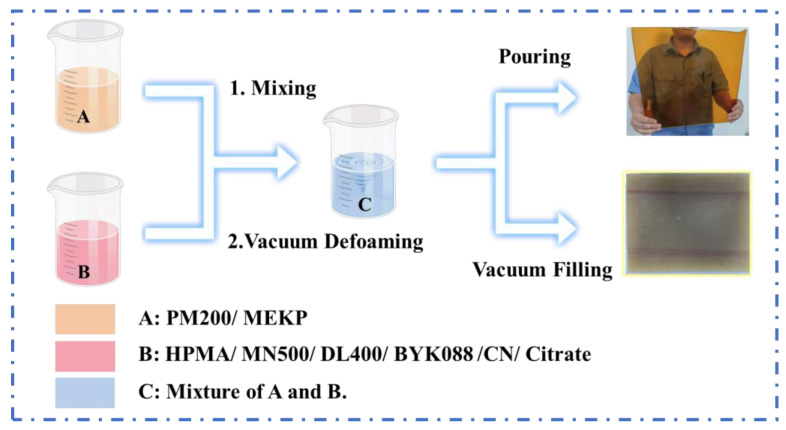
Schematic illustration of the PU/HPMA copolymer fabrication process.

Firstly, the reactor was dried at 80 °C for 3 h, and then the appropriate amounts of polymerized isocyanate MDI (PM200) and initiator methyl ethyl ketone peroxide (MEKP) were added. They were mixed and stirred under nitrogen atmosphere to obtain component A. Secondly, in another dry reactor, appropriate amounts of polyether polyol (DL400, MN 500) and HPMA were added, respectively. Stirring was applied at 80 °C for 30 min then cooled down to 30 °C. Then, appropriate amounts of defoamer BYK088, accelerator cobalt naphthenate (CN) and chelator citrate were added and stirred well to obtain component B. Components A and B were mixed to obtain mixture C and readied for processing. PU was prepared by replacing HPMA with an equal amount of polyol, which was used to form the comparison. The schematic illustration of the fabrication process is shown in Figure 1. The composition of PU samples is shown in Table 1. In order to match the integrity of the reaction, the proportions of the HPMA components were adjusted appropriately. The substrates like pouring molds and glass fiber fabrics used in this work needed to be dehydrated at 60 °C for 120 min before the process. The components involved in the reaction needed to be vacuum dehydrated before the reaction.

### 2.3. Characterization

The microscopic surface morphologies of the PU copolymers were characterized by the scanning electron microscope (SEM, NovaNano450, Hillsboro, OR, USA). Attenuated total reflection–Fourier transform infrared spectra (ATR–FTIR) of PU copolymers were obtained at room temperature using an infrared spectrometer (IS10, Thermo Nicolet, Waltham, MA, USA). The FTIR resolution, scanning range and number of scans were 4 cm^−1^, 4000–400 cm^−1^ and 32, respectively. The viscosity of the copolymers was tested under different conditions according to a rotational viscometer (Rheometer, Brookfield DV3T, Middleboro, MA, USA). The gel exothermic profile was measured by a differential scanning calorimetry (DSC, METTLER TOLEDO DSC3, Zurich, Switzerland) and a paperless recorder (PR, NHR6800, Shunchang, China). The post-curing thermal deformation process of different copolymers was recorded by thermal deformation, using a Vicat softening point tester (XRW-300UA, Beijing, China). The mechanical properties of the samples were tested by material testing machine (MST, INSTRON 5966, Canton, MA, USA). In measurement, each sample with the same size (4 mm × 10 cm × 60 cm) was stretched in the length direction with the stretching rate of 1 mm/min, and the average was calculated from 5 samples. Dynamic mechanical analysis (DMA) data were obtained with a TA Instruments Q800 instrument (Newcastle, DE, USA) at 1 Hz and 0.01% strain, from −70 to 240 °C (or until samples yielded) at a heating rate of 3 °C/min. For DMA, PU samples of approximately 20 mm × 10 mm × 4 mm were prepared by casting. 

## 3. Results and Discussion

### 3.1. Characterization of PU Copolymers

**Figure 2:**
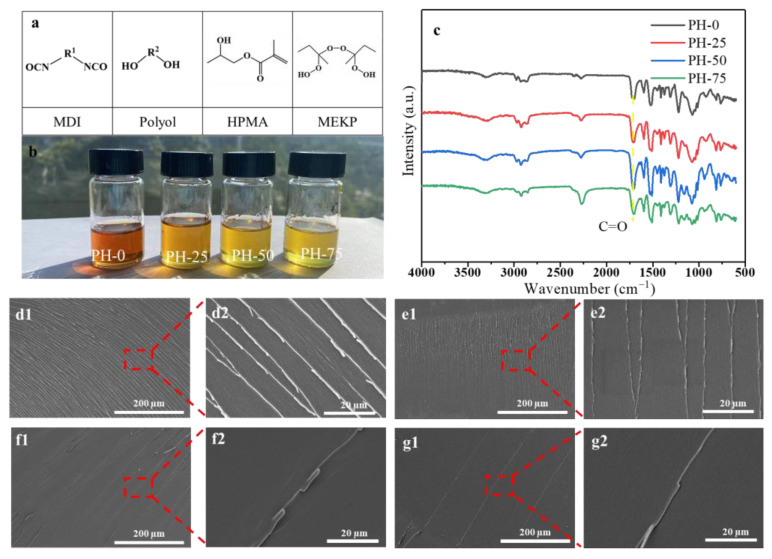
(**a**) Main compounds related to PU polymerization and radical reaction; (**b**) photographs of PH-0, PH-25, PH-50 and PH-75 mixtures; the yellow dashed line: C=O bending and vibration peak at 1713 cm^−1^; (**c**) ATR–FTIR spectrum of samples; SEM images of PH-0 (**d1**,**d2**), PH-25 (**e1**,**e2**), PH-50 (**f1**,**f2**) and PH-75 (**g1**,**g2**).

It is a complex process to introduce the free radical polymerization reaction into another [25]. In this work, we introduce a free radical polymerization reaction of HPMA into the PU reaction. The main compounds related to polymerization and radical reactions are listed in Figure 2a. Figure 2b shows the photographs of the PH-0, PH-25, PH-50 and PH-75 samples. Macroscopically, the different components of the HPMA exhibit significant color differences. As the amount of HPMA increase, the color of the mixture becomes lighter and lighter. After mixing components A and B, the mixtures are poured into the PTFE mold and cured at 80 °C for a period of time (about 4 h) to obtain various PU copolymer samples. The ATR–FTIR spectrum of samples is shown in Figure 2c. The infrared spectra of the products PH-25, PH-50 and PH-75 with the introduction of free radical polymerization are similar to PH-0. However, the introduction of HPMA increases the content of the C=O bending and vibration peak, and the intensity at 1713 cm^−1^ is significantly higher than that of PH-0 [26]. The microscopic section morphology of the samples is observed by SEM. Compared to PH-0 (Figure 2(d1,d2)), the crack structure becomes less and less in PH-25 (Figure 2(e1,e2)), PH-50 (Figure 2(f1,f2)) and PH-75 (Figure 2(g1,g2)), which may be due to the change stress required for brittle fracture after the introduction of HPMA.

### 3.2. Viscosity Curves of PU Copolymers

**Figure 3:**
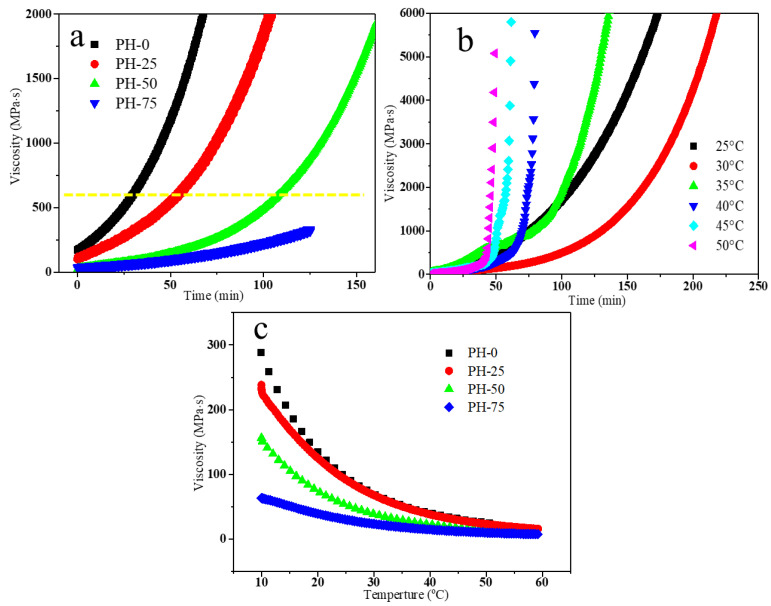
(**a**) Viscosity curves of different PU copolymers at 25 °C; the yellow hyphen line: viscosity standard (600 MPa·s) for industrial infusion; (**b**) viscosity curves of PH-50 at different temperatures; (**c**) viscosity–temperature curves of PU copolymers mixed for 1 min.

The viscosity change curves of the PU copolymers are tested by using a rheometer. In Figure 3a, the initial mixing viscosity of PU prepolymers with different components is low and the reaction is fast. It can be seen that the gelation time of PH-50 at 25 °C is about 110 min before the mix viscosity reaches 600 MPa·s, which is the viscosity standard for industrial infusion. The viscosities of PH-0 and PH-25 are too high after 50 min, and the viscosity of PH-75 is too low after 120 min during the vacuum infusion process. As the mixing temperature increasing from 25 to 50 °C, the gelation time of low viscosity PH-50 extends and then reduces in Figure 3b. The higher the mixing temperature, the lower the viscosity and the faster the reaction of the mixture due to the thermally initiated polymerization of HPMA [24]. At 30 °C, it is possible to obtain an ideal PU prepolymer for blade infusion with both low viscosity (<600 MPa·s) and a long filling time (>120 min). Figure 3c shows the ambient temperature dependence of the viscosity of the different PU copolymers after mixing for 1 min. The viscosity of the PH-50 is about 70 MPa·s at 25 °C. Even at the low temperature of 10 °C, PH-50 still maintains a low viscosity of about 180 mPa·s, which is a good performance of the infusion process. All these samples show low viscosity at different temperatures after mixing for 1 min and can be used immediately in the infusion process.

### 3.3. Gel Exothermic Behaviour and Heat Distortion of PU Copolymers

**Figure 4:**
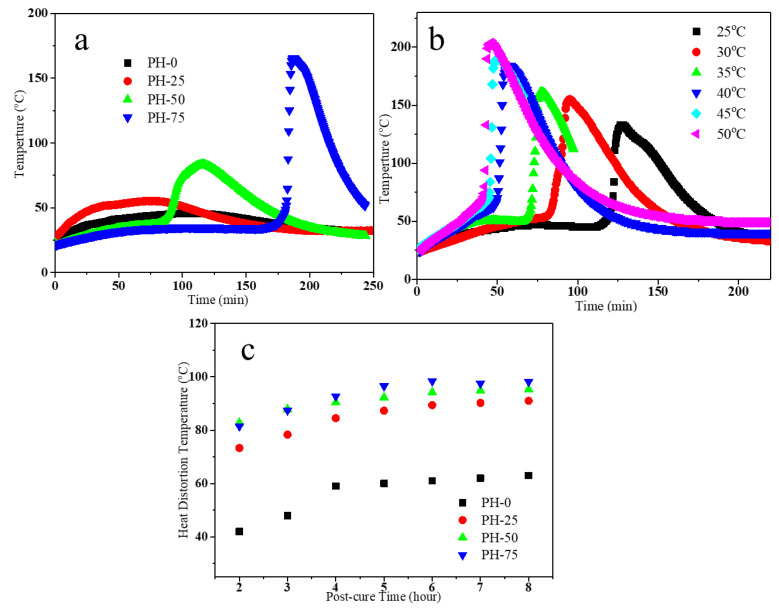
(**a**) Gel exothermic curves of different PU copolymers at 25 °C; (**b**) gel exothermic curves of PH-50 under air atmosphere; (**c**) heat deflection temperature of PU resins at post-cure temperature of 80 °C.

Figure 4a shows the gel exothermic curves of different PU samples (100 g) under air atmosphere. The exothermic temperature and gelation time both increase with increasing HPMA content. This indicates that the appropriate gelation time and temperature can be obtained by adjusting the amount of HPMA for the processing process. Figure 4b shows that the gelation time of PH-50 can be adjusted from 30 to 120 min by changing the temperature from 25 to 50 °C. Normally, with the ambient temperature at 25 °C, this sample has an effective filling time of 120 min. The low viscosity and fast flow rate can meet the infusion of large wind turbine blades. All the PU prepolymers need to be post-cured at an appropriate temperature after completing the infusion to ensure the full reaction and structure stability. Figure 4c shows the heat deflection temperature measured for the prepared PU samples at the post-curing temperature of 80 °C. It can be seen that the heat distortion temperature of PH-0, tested at 80 °C for 2 h post-curing, is only 42 °C, while the sample that underwent 4 h post-curing can reach 60 °C. However, the performance of the others is different from the PU. After curing for 2 h, the heat distortion temperatures of PH-25, PH-50 and PH-75 can all reach above 70 °C, and they can reach 80 °C after curing 4 h. The data above confirms that PH-50 has the advantages of low post-curing temperature and good heat resistance, which can improve efficiency and reduce energy consumption in the blade manufacturing process.

### 3.4. Thermal Properties of PU Copolymers

**Figure 5:**
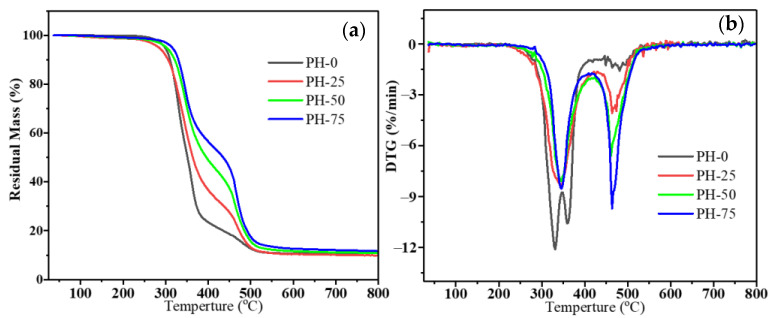
TGA (**a**) and DTG (**b**) patterns of PU copolymers under a nitrogen atmosphere.

Excellent heat resistance was critical to applicability for PU. The effect of the content of HPMA on the thermal performance of PU samples was studied. Figure 3 shows the results of the TGA and DTG referred to PU copolymers from 35 to 800 °C in nitrogen. The detail data is listed in Table 2. The *T*_5%_, *T*_30%_ and *T*_50%_ of the PU copolymers increased significantly with increasing HPMA content in Figure 5a. This indicates that the introduction of HPMA improves the thermal stability of the materials [27]. In addition, the residual mass of the copolymers increased with the addition of HPMA. In detail, the residues of PH-0, PH-25, PH-50 and PH-75 at 800 °C were 10.0 wt%, 9.8 wt%, 10.9 wt% and 11.8 wt% of the initial mass, respectively. The PH-0 sample shows two peaks at 331 and 361 °C in the DTG curve of Figure 5b. All the samples with HPMA show two sharp DTG peaks at approximately 340 and 470 °C, and these can be associated with the thermal disintegration of the matrix. These two characteristic peaks are attributed to the different amounts of PU prepolymer and HPMA. It is believed that the addition of HPMA has a good effect on the thermal stability and residual mass of the PU copolymers under an inert atmosphere.

### 3.5. Mechanical Properties of the PU Copolymers

**Figure 6:**
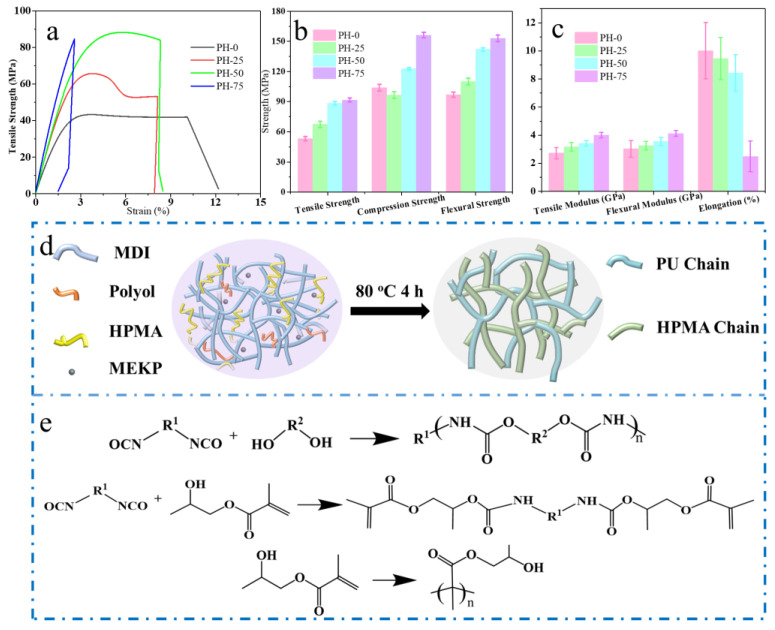
(**a**–**c**) The mechanical properties graph of PU and copolymers; (**d**) schematic diagram of mechanical properties of PU and HPMA cross-linking and (**e**) related chemical reaction formulae.

In order to verify the mechanical performance of PU copolymers, we investigated the mechanical properties of the pouring samples at room temperature. The test data are shown in Figure 6a–c, and the results are listed in Table 3. The stress–strain curves of various PU samples are shown in Figure 6a. Obviously, the PU has the highest elongation of 10.02% but the lowest tensile strength of all samples. From these data, it can be seen that the tensile strength, compression strength, bending strength and tensile modulus PU copolymers are significantly better than PH-0. Among these samples, the mechanical properties of PH-50 are the most excellent in these tests. The reinforcement of poly-HPMA to the PU is mainly attributed to the continuous cross-linking structure formed in the matrix shown in Figure 6d. The cross-linked network will limit the deformation of the matrix, making the copolymer strength and modulus have a significant increase compared to PH-0. However, this network structure is unable to withstand large strains, leading to reduced elongation. The chemical reaction formulae for PU reaction and free radical polymerization are also shown in Figure 6d. Specifically, except for the reaction between MDI and polyol that forms polyurethane chains, the isocyanate groups also react with the hydroxyl groups of HPMA in a capping reaction. At the same time, when the reaction system is heated or warmed, HPMA can react with free radical polymerization under the effect of a thermal initiator, MEKP, to form a number of poly-HPMA chains with dense cross-linking structures. In general, the addition of radical polymerization remarkably enhances the strength of the PU, indirectly improving the reliability of the PU copolymers.

**Figure 7:**
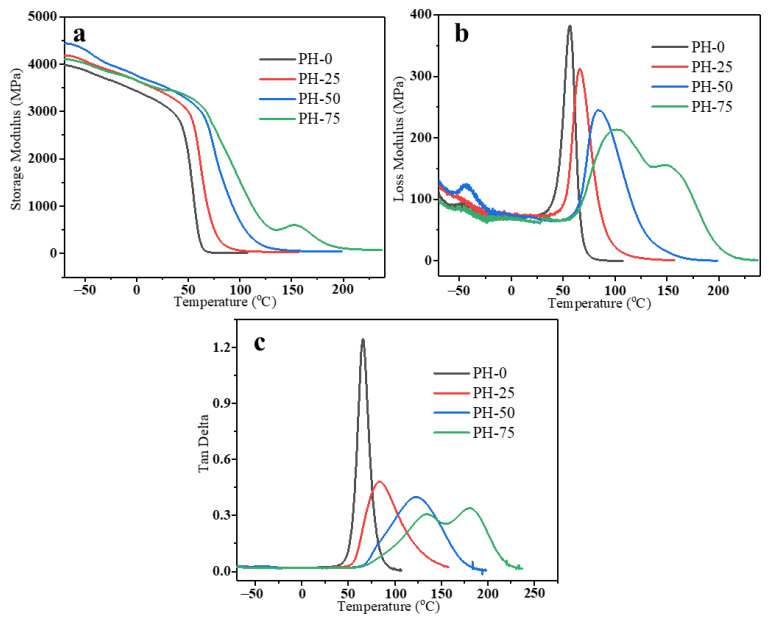
Temperature dependence of (**a**) storage modulus, (**b**) loss modulus and (**c**) tan delta for PU samples.

To further elucidate the network structure influence on dynamic stiffening, we also looked into the DMA data obtained at 1 Hz for PH-0, PH-25, PH-50 and PH-75 samples, as shown in Figure 7. In general, the storage modulus is usually related to the elasticity of the material, and it can be used to evaluate the load-carrying capacity and stiffness. In most cases, the strorage modulus of polymers depend on temperature, molecular chain structure and chain segment interactions [28,29]. Figure 7a shows that the storage modulus of the PU samples increased with an increase of HPMA content. This could be attributed to the increase in rigidity caused by the increase of HPMA and the motion limitation of the network structure. All the samples decreased continuously with increasing temperature, primarily due to the softening of the PU chains at elevated temperatures. Additionally, the storage modulus of the PH-75 sample increased slightly with the temperature rising to 155 °C. This may be due to the excess HPMA, in addition to polymerizing MDI, self-polymerizing to form a molecular chain and blend with the PU. The loss modulus is usually related to the viscosity of the material, and it can reflect the motion of the polymer chains as well as the damping due to energy dissipation. The variations of loss molulus for the PU samples as a function of temperature are shown in Figure 7b. The loss modulus of the PU copolymers containing HPMA decreased across the range of temperatures tested in comparison to that of the neat PU, which mainly resulted from the increasing of cross-linked network structure formation [30]. This network structure is not favourable for stress transmission, leading to a decrease in the loss modulus of the PU samples. The loss factor, tan delta, is the ratio of the loss modulus to the storage modulus, which usually provides key information about the mechanical properties of the composite. The glass transition temperature (Tg) of the polymer is mainly related to the delayed relaxation induced by the coordinated segmental motion of the polymer chains, which can be expressed as the temperature corresponding to the peak in the tan delta curve. As shown in Figure 7c, the Tg of PH-0, PH-25, PH-50 and PH-75 are 66, 84, 124 and 134 °C, respectively. The increase in Tg values with increasing HPMA concentration could be mainly attributed to the network structure that limited the mobility of the PU chains. In addition, it was clearly seen that the peak value of tan delta for PU-HPMA decreased, suggesting that the PU chains’ mobility decreased after being doped with HPMA.

### 3.6. SEM Image of PU Copolymers with Glass Fiber Fabric

**Figure 8:**
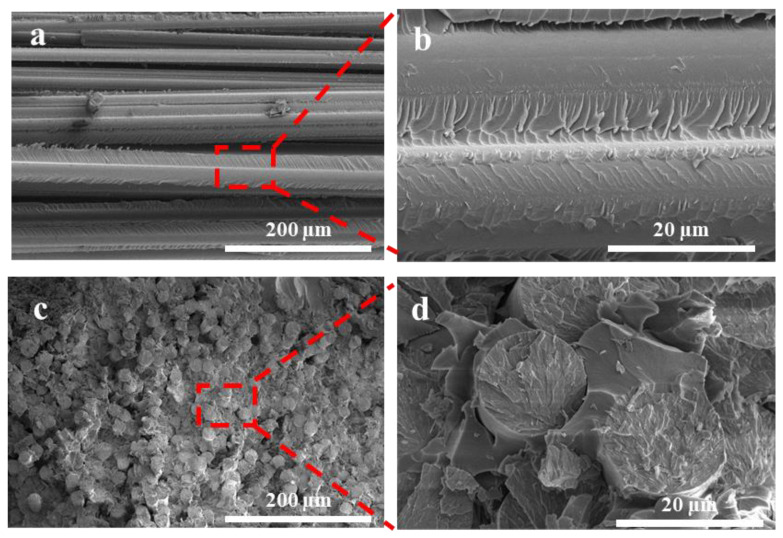
Cross-section SEM micrographs of PH-50/E7-UD1200 composite (**a**,**b**) in the 90° direction and (**c**,**d**) in the 0° direction.

The data above shows that the PH-50 has the best processing characteristics and mechanical properties. Therefore, we fabricated a PH-50/GFF composite with excellent performance by vacuum infusion of the PH-50 and many types of glass fiber fabrics for use in different positions in wind turbine blades, such as the E6-UD1200, E7-UD650 and E8-UD1250. The morphologies of the PH-50/E7-UD1200 composites were evaluated with scanning electron microscopy (SEM). Figure 8a,b show the SEM micrographs of the PH-50/E7-UD1200 by vacuum infusion in the 90° direction. Figure 8c,d show them in the 0° direction. Figure 8a reveals the cross-section of the 0° sample is smooth and relatively flat. Obvious defects or delamination cannot be found between the PH-50 and glass fiber fabric in Figure 8b. This demonstrates that the distribution of glass fiber in the PH-50 is relatively uniform. The low and high magnified cross-section SEM images in Figure 8c,d in the 0° direction reveals that the glass fiber fabrics are evenly surrounded by the PH-50 matrix without agglomeration. A significant interface can not be observed between the PH-50 matrix and the glass fiber fabric, proving that the two materials are very compatible. Therefore, the vacuum-infused PH-50/GFF composites are morphologically stable and have great potential to be used as wind turbine blade materials. 

### 3.7. Mechanical Properties of Vacuum-Infused PU/GFF Composites

**Figure 9:**
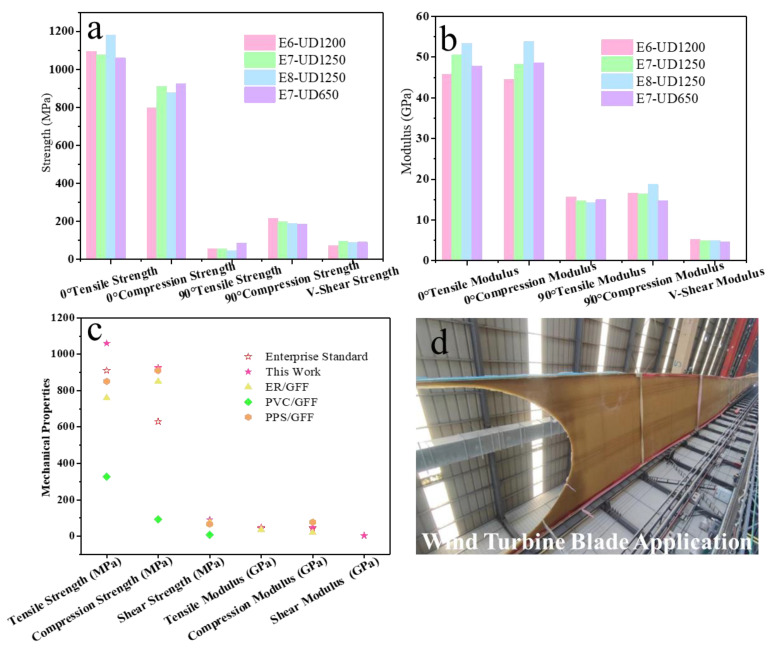
(**a**) The mechanical strength and (**b**) modulus graphs of PH-50/GFF composite; (**c**) mechanical properties of enterprise standard and different polymer composites; (**d**) application of PH-50/GFF composite in wind turbine blade.

In order to further verify the comprehensive performance of the vacuum-infused PH-50/GFF composites, the mechanical properties of the samples are tested using the material testing machine. The results of the strength, modulus and strain of the PH-50/GFF composites are shown in Figure 9a,b and Table 4. As shown in Figure 9a, the tensile strengths of all the PU-50/GFF, made from four types of glass fibers fabrics, in the 0° direction (parallel to the glass fiber direction) are above 1000 MPa and, in the 90° direction (perpendicular to the glass fiber direction), are all above 52 MPa. The compression strength in the 0° direction are above 800 MPa and, in the 90° direction, are above 187 MPa. Furthermore, all these composites have high tensile moduli and compression moduli, shown in Figure 9b, which fully meet the current design requirements for wind turbine blades. The mechanical properties of different polymer composites and enterprise standard are shown in Figure 9c and Table 5. The strength and modulus of the PU/GFF composite are higher than that of other polymer composites like epoxy resin (ER), polyvinyl chloride (PVC), polyphenylene sulfide (PPS) and others. The merits of the present composites are extremely high mechanical strength and an excellent modulus, which can meet the requirement of wind turbine blade composites [31,32,33]. The application of PH-50/GFF composites in wind turbine blades is shown in Figure 9d. Different types of PH-50/GFF composites may be applied to different types of wind turbine blades or to different locations on the same blade. Until now, this composite has achieved successful application in a blade type, and the prepared wind turbine blade has gained relevant approvals. We suspect that these composites will be used on a large scale for wind turbine blades in the future.

## 4. Conclusions

In this work, we construct a dense cross-linking structure in polyurethane (PU) based on different amounts of hydroxypropyl methacrylate (HPMA) to achieve low processing viscosity and excellent mechanical properties. The HPMA monomers significantly reduce the viscosity of the mixing gel, prolong the gelation time and increase the cross-linking structure. The PU prepolymer exhibits a low viscosity and a long gelation time at 25 °C, which is sufficient to complete processes like pouring or filling. After the pouring and curing process, the PU copolymer shows excellent mechanical and dynamic thermo-mechanical properties due to the cross-linking structure between the PU chains and the poly-HPMA chains. Additionally, the PU samples show excellent compatibility with various glass fiber fabrics (GFF) in the vacuum-infusion experiment. The PU/GFF composites have high strengths and moduli for wind turbine blades in various positions. Specifically, the tensile strengths of the PU/GFF composites are all over 1000 MPa in the 0° direction. Such copolymers and composites are promising for the future production of wind turbine blades that meet the stringent requirements for outstanding processing and mechanical properties.

## Figures and Tables

**Table 1:** Composition of the PU samples (200 g copolymer).

Component	A		B					
Samples	PM200	MEKP	HPMA	MN500	DL400	BYK088	CN	Citrate
PH-0	99	1	0	60	39.5	0.25	0.15	0.1
PH-25	99	1	25	45	29.5	0.25	0.15	0.1
PH-50	99	1	50	30	19.5	0.25	0.15	0.1
PH-75	99	1	75	15	9.5	0.25	0.15	0.1

**Table 2:** TGA data of samples under a nitrogen atmosphere.

Samples	T_5%_ (°C)	T_30%_ (°C)	T_50%_ (°C)	T_peak1_ (°C)	T_peak2_ (°C)	Residue at 800 °C (wt%)
PH-0	298.9	331.0	351.5	331.0	361.0	10.0
PH-25	283.9	338.2	363.9	345.0	464.0	9.9
PH-50	302.2	350.8	395.5	345.0	461.0	10.9
PH-75	315.6	356.1	435.0	345.0	463.0	11.8

**Table 3:** Mechanical and physical properties of the cast copolymers.

Samples	Tensile Strength (MPa)	Tensile Modulus (GPa)	Elongation at Break (%)	Compression Strength (MPa)	Flexural Modulus (GPa)	Flexural Strength (MPa)
PH-0	53.3	2.7	10.0	104.0	3.0	97.0
PH-25	67.5	3.2	9.5	110.0	3.3	110.0
PH-50	83.4	3.4	8.4	120.0	3.6	142.0
PH-75	91.7	4.0	2.5	153.0	4.1	153.0

**Table 4:** Mechanical and physical properties of PH-50/GFF composites.

Samples	PH-50/ E6-UD1200	PH-50/ E7-UD1250	PH-50/ E8-UD1250	PH-50/ E7-UD650
0° Tensile Strength (MPa)	1094.2	1077.1	1182.0	1060.4
0° Compression Strength (MPa)	799.8	912.1	876.9	926.1
90° Tensile Strength (MPa)	57.8	54.9	46.9	87.3
90° Compression Strength (MPa)	217.4	200.0	189.1	187.1
V-Shear Strength (MPa)	74.3	95.8	89.5	92.2
0° TensileModulus (GPa)	45.8	50.6	53.4	48.7
0° Compression Modulus (GPa)	44.5	48.2	53.8	48.6
90° TensileModulus (GPa)	15.7	14.7	14.3	19.5
90° Compression Modulus (GPa)	16.6	16.5	18.7	15.1
V-ShearModulus (GPa)	5.2	4.9	5.0	4.6

**Table 5:** Mechanical properties of enterprise standard and different polymer composites.

Samples	Enterprise Standard	PU/GFF	ER/GFF	PCV/GFF	PSS/GFF
Tensile Strength (MPa)	910	1077.1	760	328	851
Compression Strength (MPa)	630	912.1	850	93.3	911
V-Shear Strength (MPa)	-	95.8	75	8	67
0° TensileModulus (GPa)	43.5	50.6	36	-	-
0° Compression Modulus (GPa)	43.5	48.2	20	-	78
V-ShearModulus (GPa)	3.8	4.9	-	-	-

## Data Availability

Data from this study are available upon request from the corresponding authors.
